# Reduced FAF1 Expression and *Helicobacter* Infection: Correlations with Clinicopathological Features in Gastric Cancer

**DOI:** 10.1155/2012/153219

**Published:** 2012-12-11

**Authors:** Ai-qun Liu, Lian-ying Ge, Xin-qing Ye, Xiao-ling Luo, Yuan Luo

**Affiliations:** ^1^Department of Endoscopy, The Affiliated Tumor Hospital Guangxi Medical University, Nanning, Guangxi 530021, China; ^2^Department of Pathology, The Affiliated Tumor Hospital Guangxi Medical University, Nanning, Guangxi 530021, China; ^3^Science and Technology Agency, Guangxi Medical University, Nanning, Guangxi 530021, China

## Abstract

Background. This study aimed to investigate possible associations between FAF1 expression and aspects of gastric cancer, in particular its clinical characteristics and *Helicobacter* infection. Materials and Methods. RT-PCR and immunohistochemistry were used to analyze expression of FAF1 mRNA and protein in 40 gastric cancer patients.* H. pylori *infection was detected by three staining protocols. Results. The expression level of FAF1 mRNA was significantly lower in gastric cancer tissue than in normal gastric mucosa from the same patient (*P* < 0.05). FAF1 mRNA expression was significantly lower in stage IV gastric cancer than in stage I+II or IIIA+IIIB (*P* = 0.004) and also significantly lower in gastric cancer with distant metastasis. FAF1 mRNA expression was higher in well-differentiated cancer than in poorly-differentiated cancer (0.39 ± 0.06 versus 0.19 ± 0.06, *t* = 9.966, *P* < 0.01). FAF1 protein was detected in 15 of 40 (37.5%) cancerous tissue samples and in 29 of 40 (72.5%) corresponding normal tissue samples (*P* < 0.01). FAF1 mRNA expression was lower in *H. pylori*-positive cancerous tissue samples than in *H. pylori*-negative ones (*P* < 0.05). Conclusions. Downregulation of FAF1 expression may be related to the carcinogenesis and progression of gastric cancer, and *H. pylori* infection during gastric carcinogenesis may downregulate FAF1 expression.

## 1. Introduction

Recently Fas-associated factor 1 (FAF1) was discovered and characterized as a potent regulator of cell survival, facilitating apoptosis by interacting with Fas-associated death domain (FADD), caspase-8, and protein kinase CK2-*β* [1–3]. It is a component of the death-inducing signaling complex, and when overexpressed, it can initiate apoptosis and induce cell death in some cell types even in the absence of any extrinsic death signals [1]. FAF1 binds death effector domain-interacting domain (DEDID), and it interacts with the death domain of Fas through its Fas-interacting domain (FID) [4, 5].

Subsequent work has revealed that the FID of FAF1 interacts with a variety of downstream targets, and functional loss of FAF1 may provide a prosurvival signal to cells in disease states such as cancer [6]. In fact, loss or downregulation of FAF1 expression has been observed in various human cancers [7, 8]. Western blotting studies have shown that FAF1 protein levels are lower in human gastric carcinoma tissue than in healthy control tissue from the same patients [9]. However, it remains unclear whether the lower protein levels reflect lower levels of FAF1 gene expression, and whether that downregulation is associated with the clinical characteristics of gastric cancer, including the often-observed comorbidity of gastric infection by *Helicobacter pylori*. 

Gastric carcinoma is a major cause of morbidity and mortality worldwide. The precise mechanism of gastric carcinogenesis is not yet fully understood. *H. pylori* is a Gram-negative bacillus capable of colonizing the gastric mucosa. Countries with high rates of gastric cancer, such as China and Japan, tend to have a high prevalence of *H. pylori* infection [10]. Although *H. pylori* infection is considered one of the earliest steps in gastric carcinogenesis, how it contributes to the disease remains obscure [11, 12].

In the stomach, a homeostatic balance is maintained between the proliferation and apoptosis of gastric mucosal cells, and changes in this balance seem to be the biological basis of gastric carcinogenesis [13]. There is increasing evidence that apoptosis plays an important role in the pathogenesis of a variety of diseases caused by bacteria, viruses, and other factors; apoptosis is also regarded as one of the most important mechanisms of tumor cell suicide [14, 15]. The process of apoptosis involves a wide variety of regulatory and effector molecules, and Fas is one of the most important groups of apoptosis regulators [16]. 

Increasing evidence indicates that bacterial pathogens modulate the apoptotic signaling cascade of host cells and thereby cause disease [15]. However, we are unaware of studies examining whether *H. pylori* infection is associated with changes in FAF1 expression during gastric carcinogenesis. In the present study, we investigated this and other possible associations between clinical characteristics of gastric cancer and FAF1 expression.

## 2. Methods

### 2.1. Patients and Design

This study involved 40 patients with gastric cancer (29 male, 11 female) who were referred for surgery between June 2005 and April 2006. Patients had either been admitted directly to the Affiliated Tumor Hospital of Guangxi Medical University, or they had been admitted first to the People's Hospital of Guangxi Zhuang Autonomous Region and then referred to the Affiliated Tumor Hospital. Patient age ranged from 34 to 78 years (median 55). None received neoadjuvant treatments. Prior informed consent was obtained, and the study protocol was approved by the Ethics Committees of both the Affiliated Tumor Hospital and the People's Hospital. 

Tissue specimens from both the cancerous lesion and from normal gastric mucosa located more than 5 cm from the primary tumor were collected from each patient at the time of surgery. Specimens were immediately frozen in liquid nitrogen, and stored at −80°C until use. All cases were reviewed by two specialists to confirm diagnosis according to the criteria of the Japanese Gastric Cancer Association [17]. 

Specimens of paracancerous tissue, defined as noncancerous gastric tissue located less than 2 cm from the primary tumor, were collected at the time of surgery, fixed in 10% neutral formalin, and embedded in paraffin. Serial thin sections were prepared and placed on glass slides coated with 3-aminopropyltriethoxysilane (Sigma, USA), then stained as described below to detect FAF1 protein and *H. pylori* infection. 

### 2.2. cDNA Synthesis and RT-PCR

Gastric tissue specimens (approximately 0.05–0.1 g) were homogenized with a homogenizer, and total RNA was extracted with a Trizol kit (Invitrogen, USA). Total RNA (3 *μ*g) from each sample was reverse-transcribed into cDNA using the AMV Reverse Transcription system (Promega, USA). As a reference for normalizing levels of FAF1 mRNA, part of the *β*-actin housekeeping gene was amplified using 2 *μ*L of cDNA from each sample. 

Primers were designed using Primer Premier 5.0 (Premier Biosoft International, USA) based on published mRNA sequences of FAF1 (GenBank NM_007051.2) and *β*-actin (NM_001101). The sequences of the PCR primers were: FAF1, 5′-cttgctgaatcagggctctc-3′ (Forward) and 5′-tccaccccaaattctgtagc-3′ (Reverse) to give a 164-bp product; and *β*-actin, 5′-accgagcgcggctacagc-3′ (Forward) and 5′-ctcattgccaatggtgat-3′ (Reverse) to give a 180-bp product. 

RT-PCR was performed using a SYBR Green kit (Applied Biosystems, USA) in an ABI Prism 7300 HT Sequencer (PE Applied Biosystems, USA) according to the manufacturer's instructions. The following cycling parameters were used: 5 min at 94°C; 35 cycles of 45 s at 94°C, 45 s at 55°C and 1 min at 72°C; and finally 10 min at 72°C. Levels of FAF1 mRNA were calculated based on the threshold cycle (CT) values and normalized to levels of *β*-actin mRNA. The results were analyzed using the 2^−Δ*C*_*T*_^ method and the formula Δ*C*_*T*_ = Avg · FAF1  *C*_*T*_ − Avg · *β*-actin  *C*_*T*_. 

### 2.3. Immunohistochemistry

FAF1 protein was detected by immunohistochemistry using a monoclonal anti-FAF1 antibody (clone 1A10, Santa Cruz Biotechnology, USA) at a 1 : 100 dilution. Antibody staining was carried out strictly according to the manufacturer's instructions. Paraffin sections of rat kidney served as a positive control. As negative controls, experimental patient specimen slides were prepared and subjected to the normal antibody staining procedure but with the primary antibodies omitted. Immunolabelling was detected using an avidin-biotin complex and 3,3′-diaminobenzidine as chromogen (Fuzhou Maixin Biotechnology Development Co. Ltd., China). Sections were counterstained with hematoxylin. 

### 2.4. Evaluation and Scoring of FAF1-Positive Cells

For each patient, four peripheral fields and a middle field were selected, and the numbers of cells positive for FAF1 protein were determined for a sample of 500 tumor cells and for 500 corresponding normal gastric mucosa cells. Slides were semiquantitatively evaluated by two pathologists working independently (Y. Luo and X.-q. Ye). If the percentage of cells positive for FAF1 protein was <6% of the 500 cells examined, then the sample was judged to be negative for FAF1 protein expression; if the percentage was ≥6%, it was considered positive. Average values were determined for all cancerous tissue samples and noncancerous tissue samples.

### 2.5. Detection of *H. pylori* Infection

Samples of normal gastric tissue, paracancerous tissue, and primary tumor tissue were taken from each patient. To reduce the risk of false positives, the method of Madan et al. [18] was used. Briefly, thin sections (4 *μ*m) from each tissue sample were stained with hematoxylin and eosin (H&E), toluidine blue, and Warthin-Starry silver in order to detect *H. pylori* infection. Samples had to be positive by at least two staining methods in order to be judged *H. pylori*-positive. Samples were divided into a subgroup of *H. pylori*-negative cancerous samples and a subgroup of *H. pylori*-positive cancerous samples.

### 2.6. Statistical Analysis

A database of clinicopathological information on the 40 patients was set up in SPSS 16.0 (SPSS, Inc., USA), which was used to analyze the data for associations and conduct all statistical tests. Measurements are presented as the mean ± SD. Differences in FAF1 mRNA expression between gastric cancer tissue samples and the corresponding normal gastric mucosa samples were examined using the paired *t*-test and a test for homogeneity of variance. Differences in FAF1 mRNA expression between the cancerous and normal groups on one hand and clinicopathological features on the other were examined using the independent-samples *t* test and one-way ANOVA. Differences in percentages of cells positive for FAF1 protein were examined using *χ* ^2^ analysis. *P* < 0.05 was considered statistically significant. 

## 3. Results 

### 3.1. Expression of FAF1 mRNA and Protein in Gastric Cancer

**Figure 1:**
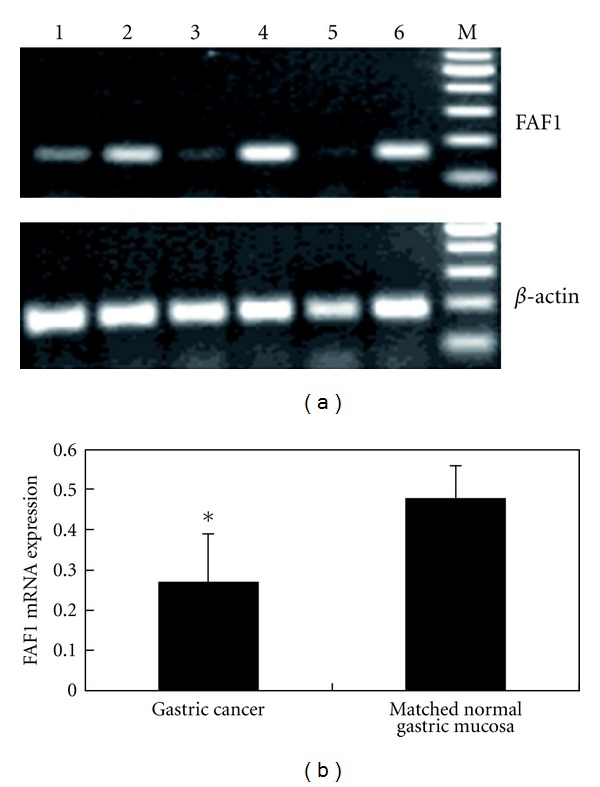
RT-PCR analysis of FAF1 mRNA expression in gastric cancer tissue and matched normal gastric mucosa tissue. (a) RT-PCR to measure levels of FAF1 and *β*-actin mRNA. Lanes 1, 3, 5: gastric cancer tissue from different patients. Lanes 2, 4, 6: corresponding normal gastric mucosa tissue from the same patients. M, 100-bp DNA marker ladder. *β*-actin was used as an internal control. (b) Comparison of FAF1 mRNA levels (normalized to *β*-actin levels) in gastric cancer tissue and matched normal gastric mucosa tissue. FAF1 mRNA levels were much lower in the gastric cancer tissue samples based on the mean ± SD test. *P < 0.05.

**Figure 2:**
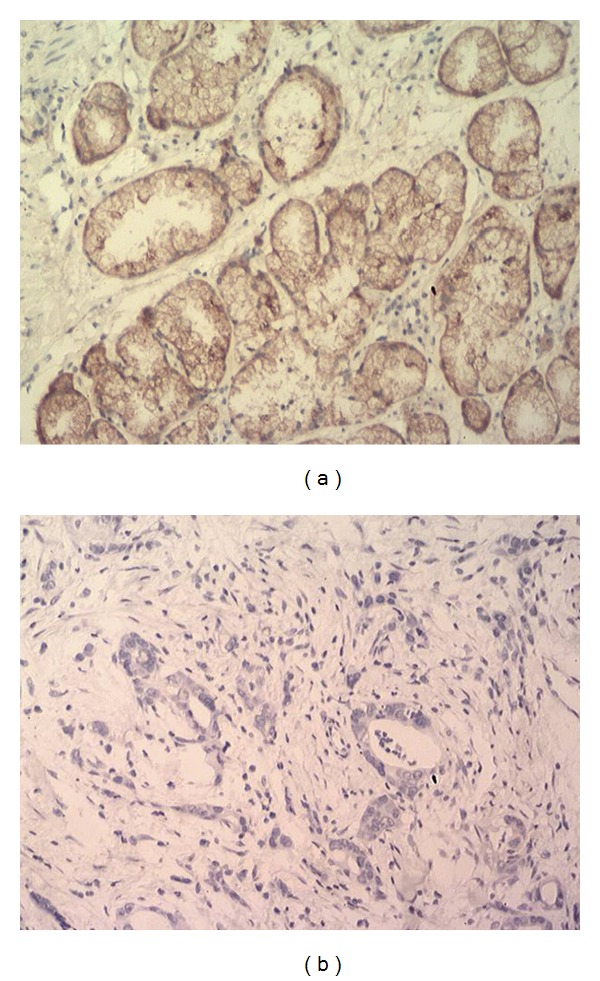
Immunohistochemistry to detect FAF1 protein. (a) Normal gastric mucosa tissue and (b) gastric cancer tissue from the same patient. FAF1 staining localized mainly to the nucleus and in some cases to the cell membrane. Magnification, 200x.

Expression of FAF1 mRNA and *β*-actin mRNA was detected in all 40 gastric cancer samples and corresponding normal gastric mucosa samples. FAF1 mRNA expression was significantly lower in gastric cancer samples than in the matched normal gastric mucosa samples (0.27 ± 0.12 versus 0.48 ± 0.08, *t* = 9.209,  *P* < 0.05) (Figure 1). Consistent with this finding, FAF1 protein was detected in only 15 of 40 (37.5%) gastric cancer samples, compared to 29 of 40 (72.5%) corresponding normal gastric mucosa samples (*χ* ^2^ = 9.899, *P* < 0.01). FAF1 protein staining localized primarily to the nucleus and in some cases to the cell membrane (Figure 2). 

 We performed various subgroup analyses to examine possible associations between FAF1 mRNA expression and clinicopathological features of gastric cancer. We examined patient gender, age, size, histological grade, invasion depth, lymph node metastasis, distant metastasis, and clinical staging. FAF1 mRNA expression level was lower in stage IV gastric cancer tissue (0.18 ± 0.12) than in Stage I + II (0.32 ± 0.12) or stage IIIA + IIIB cancer tissue (0.30 ± 0.11, *F* = 6.276, *P* = 0.004), and it was lower in gastric cancer with distant metastasis than in gastric cancer without distant metastasis (0.19 ± 0.07 versus 0.29 ± 0.12, *t* = −2.753, *P* < 0.01). Conversely, the FAF1 mRNA expression level was higher in well-differentiated cancer tissue than in poorly differentiated cancer tissue (0.39 ± 0.06 versus 0.19 ± 0.06, *t* = 9.966, *P* < 0.001). FAF1 mRNA expression showed no obvious association with gender, age, tumor size, infiltration degree, lymph node metastasis or clinical stage below IV in gastric cancer (Table 1). 

### 3.2. Prevalence of *H. pylori* Infection

**Figure 3:**
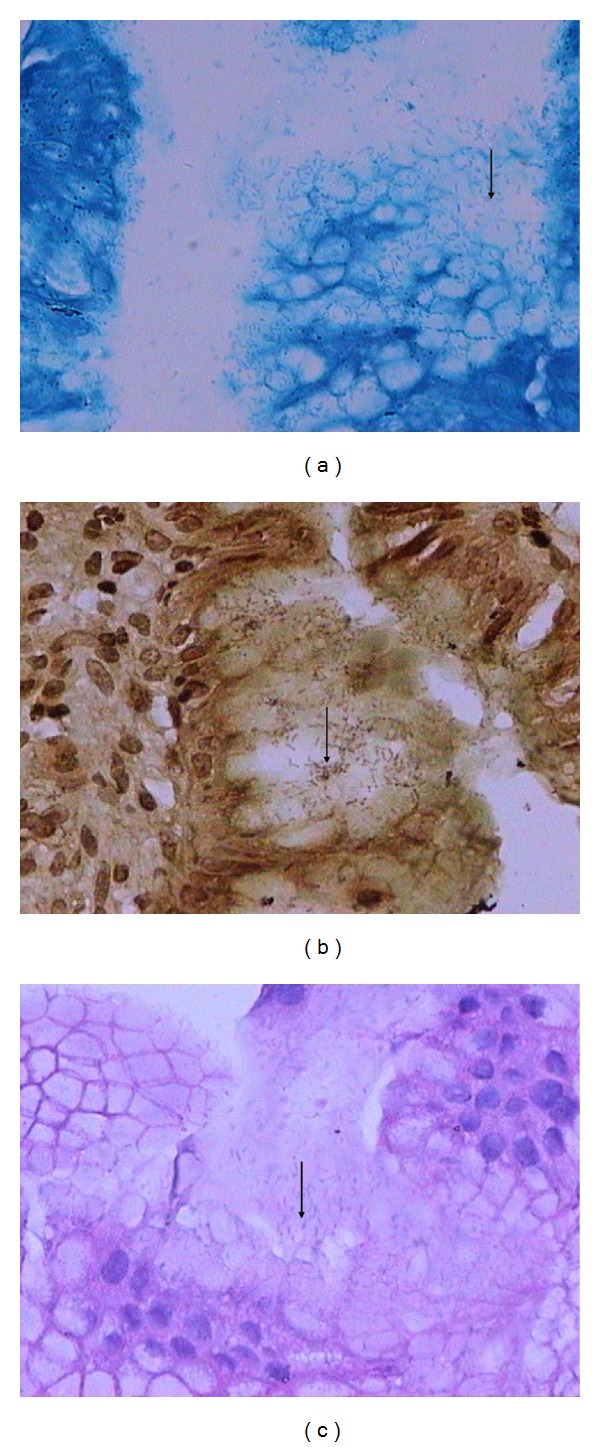
*H. pylori* aggregation on the surface of gastric mucosa. *H. pylori* was detected as (a) light blue staining using toluidine blue, (b) brownish-black staining using Warthin-Starry silver, and (c) light purple using H&E. Arrows indicate *H. pylori *bacilli. Magnification, 400x.

*H. pylori* was detected by three staining methods: it appeared light purple by H&E, brownish-black by Warthin-Starry silver and light blue by toluidine blue (Figure 3). *H. pylori* was found to exist primarily in the paracancerous tissue; it was rarely found in necrotic cancer tissue, perhaps because the microenvironment was unsuitable for its growth. It was usually found clustered in glandular organ cryptae of gastric mucosa. Based on H&E staining, 25 of 40 (62.5%) biopsies were positive for *H. pylori,* including 6 biopsies negative by Warthin-Starry silver staining and 5 negative by toluidine blue. Only 1 biopsy was positive by Warthin-Starry silver and toluidine blue, but negative by H&E. Applying the criterion that biopsies must be positive by at least two staining methods to be considered positive for *H. pylori,* our results indicate an infection rate of 21 of 40 (52.5%).

### 3.3. Association between FAF1 mRNA Expression and *H. pylori* Infection

FAF1 mRNA expression was lower in *H. pylori*-positive tissue samples than in *H. pylori*-negative samples from gastric cancer patients (0.18 ± 0.06 versus 0.29 ± 0.12, *t* = 3.6084, *P* < 0.05). In contrast, among normal gastric mucosa samples from the same patients, FAF1 mRNA expression did not vary with the presence or absence of *H. pylori* infection (0.49 ± 0.08 versus 0.47 ± 0.11, *t* = 0.6515, *P* > 0.05).

## 4. Discussion

The recurring loss or downregulation of FAF1 expression observed in certain human and murine cancers suggests that this proapoptotic factor is a tumor suppressor, though FAF1 expression has yet to be analyzed in detail in several other human cancers, including gastric cancer [19, 20]. Some authors have reported longer survival in Fas-positive cancer patients, suggesting that high Fas expression may inhibit tumor growth and implying that malfunction of Fas-dependent apoptotic pathways may be associated with more aggressive tumor formation and shorter survival [21]. FAF1 is a Fas-binding protein that negatively regulates capsaicin-induced apoptosis of cancer cells [22]. In the present study, we found that expression of both FAF1 mRNA and protein was reduced in gastric cancer tissue, and the level of FAF1 mRNA expression was associated with tumor differentiation, distant metastasis, and clinical stage of tumors. 

Bjørling-Poulsen et al. [9] used Western blotting to show that levels of FAF1 protein were lower in gastric cancer tissue than in noncancerous tissue. Since protein levels are only an indirect measure of gene expression, we wished to probe directly whether FAF1 gene expression was altered during gastric cancer. Therefore we carried out RT-PCR studies in combination with immunohistochemistry to gain a more comprehensive picture of gene and protein regulation. Our results confirm the work of Bjørling-Poulsen et al. [9]. They further establish RT-PCR detection of FAF1 mRNA as a reliable and sensitive technique that may prove useful as a novel diagnostic tool for gastric cancer. 

While the role of FAF1 in apoptosis is well known, its involvement in cell differentiation is less clear. At the very least, it is expected to play a key role, since FAF1-deficient mouse embryos die around the 2-cell stage, indicating that FAF1 is essential for cell viability and/or cell division [23]. FAF1 has been reported to act as a negative regulator of Wnt/beta-catenin signaling, which plays an important role in the regulation of cell proliferation and differentiation [24]. Given the proapoptotic function of FAF1, downregulation would be expected to promote the survival of tumor cells as well as their resistance to anticancer therapy [6]. In fact, FAF1 has recently been shown to mediate chemotherapy-induced apoptosis by promoting the formation of death effector filaments (DEFs), structures associated with receptor-independent apoptosis [25]. These findings may mean that increasing FAF1 expression or activity would be a good therapeutic goal.

Metastasis is a complex process involving degradation of the basement membrane, invasion of the stroma, adhesion, angiogenesis, and cell proliferation and migration. Our findings suggest that reduced FAF1 expression is associated with gastric cancer metastasis. This association may be related to recent research describing a role for miRNAs in biological pathways related to metastasis, including angiogenesis and apoptosis [26]. MiR-24 has been reported to target the FAF1 gene in the prostate cancer cell line DU-145 [27]. It would be interesting to investigate whether MiR-24 helps to explain the observed association between FAF1 gene downregulation and gastric cancer metastasis. 

We also found FAF1 gene expression to be much lower in stage IV gastric cancer than in earlier stages of the disease. This may reflect the same processes behind the association between FAF1 downregulation and metastasis, since metastasis is indispensable for aggressive development of gastric cancer. The subgroup analyses by metastasis and clinical staging suggest that FAF1 acts as a tumor suppressor and its downregulation contributes to gastric cancer onset and progression. On the other hand, we found that FAF1 mRNA expression was higher in well-differentiated cancer tissue than in poorly differentiated cancer tissue. In addition, FAF1 mRNA expression showed no obvious association with tumor size, infiltration degree, lymph node metastasis or clinical stage below IV. It is possible that we failed to detect associations between FAF1 expression and clinicopathological features of gastric cancer because of our small sample size, in particular in our subgroup analyses, in which the smallest subgroup had only 3 patients. Therefore our findings require evaluation in future studies with larger sample sizes. 

Various tools have been employed to identify the association between *H. pylori* and gastric cancer. Gene expression analysis using cDNA microarrays has shown that *H. pylori* infection significantly alters the expression of genes related to apoptosis and proliferation in human gastric carcinoma cells [28, 29]. However, we are unaware of reports probing the possible association between FAF1 gene expression and *H. pylori* infection in cancerous and noncancerous gastric mucosa. In our study, we used a strict triple-staining approach to detect *H. pylori* infection, which was found in 21 of 40 (52.5%) gastric cancer patients. This frequency is lower than the 171 of 214 cases (80%) reported by Motta et al. [30]. The reason for the discrepancy may be due to the smaller number of patients in our study, or to the fact that we applied a stricter screening method. These findings suggest that *H. pylori* infection may contribute to the downregulation of FAF1 gene expression during gastric carcinogenesis, and that this may contribute to malignant transformation. However, since a substantial percentage of our patients did not show the presence of *H. pylori*, our results suggest that the pathogen is not a prerequisite for onset or progression of gastric cancer.

Instead, the findings of our studies and others suggest that the development of gastric cancer is a multifactorial process in which *H. pylori* can participate but does not have to. Several studies have suggested plausible ways in which *H. pylori* infection may contribute to gastric cancer. The bacteria can induce Fas Ag- and ligand-mediated apoptosis, either directly or indirectly via cytokines IL-1*β*, TNF*α* and IFN*γ* [31]. Increased apoptosis following *H. pylori* infection leads to gastric atrophy, while inhibition of apoptosis results in cell proliferation and transformation into cancer. If this occurs early during infection and persists, gastric cells can become resistant to Fas-mediated apoptosis [32, 33]. The Fas pathway may then drive cell proliferation and cell turnover. *H. pylori* can also bind to MHC-II in cultured cells and inhibit Fas Ag-mediated apoptosis [34]. In the present study, we found evidence for an additional effect of *H. pylori* infection: FAF1 mRNA expression was lower in *H. pylori*-positive tissue samples than in *H. pylori*-negative samples from gastric cancer patients. This result, moreover, was specific to cancerous tissue: the presence or absence of *H. pylori* did not affect the levels of FAF1 mRNA in noncancerous mucosa samples from the same gastric cancer patients. Our results suggest that *H. pylori* infection inhibits FAF1 mRNA expression, but this finding remains speculative. Future studies should examine larger samples sizes and should compare samples from gastric cancer patients and control samples from age- and sex-matched healthy individuals. 

Our study was limited by a relatively small number of patients. In particular, subgroups were small, affecting the reliability of our analyses. In this study, we did not evaluate a possible association between FAF1 protein levels and *H. pylori* infection, which requires further study. Our work was also unable to identify how FAF1 activity may contribute to gastric cancer onset and progression. FAF1 has multiple protein-interacting domains and it may function in several signal transduction pathways [35, 36], so careful mechanistic studies are needed. We postulate that loss of FAF1 function may have far-reaching effects in cancer, although its role(s) may be context-dependent. Larger, more extensive studies are needed to understand better the role of FAF1 in signaling pathways vital to both normal development and tumorigenesis in *H. pylori* infection.

## 5. Conclusions

Expression of FAF1 mRNA and protein was lower in gastric cancer samples than in normal gastric mucosa samples from the same patients. Downregulation of FAF1 mRNA was associated with tumor differentiation and distant metastasis. *H. pylori* may downregulate FAF1 expression in gastric carcinogenesis. Consequently, increasing FAF1 expression through gene therapy may be effective for treating gastric cancer in the future. 

## Figures and Tables

**Table 1:** FAF1 mRNA expression and clinicopathological characteristics of gastric cancer.

Parameter	*n*	FAF1 mRNA level**	*t*(*F*)	*P*
Gender				
Female	11	0.27 ± 0.12	0.192	0.849
Male	29	0.26 ± 0.11		
Age (years)				
≥60	17	0.27 ± 0.12	0.53	0.599
<60	23	0.25 ± 0.11		
Tumor size (cm)*				
≥5	18	0.26 ± 0.11	0.1	0.921
<5	22	0.26 ± 0.12		
Histological grade				
Well differentiated	14	0.39 ± 0.06	9.966	<0.001
Poorly differentiated	26	0.19 ± 0.06		
Invasion depth				
No serosa invasion	7	0.30 ± 0.12	1.09	0.283
Serosa invasion	33	0.25 ± 0.11		
Lymph node metastasis				
Present	3	0.34 ± 0.14	1.266	0.213
Absent	37	0.25 ± 0.11		
Distant metastasis				
Present	12	0.19 ± 0.07	−2.753	0.009^a^
Absent	28	0.29 ± 0.12		
Clinical stage				
I + II	8	0.32 ± 0.12	6.276	0.004^a^
III_A_ + III_B_	17	0.30 ± 0.11		
IV	15	0.18 ± 0.12		

*Defined by the longest dimension in the gastric mucosa.

**Normalized to the level of *β*-actin.

^a^ *P* < 0.01, based on a comparison using a mean ± SD test.
